# Non-invasive Approaches for the Diagnosis of Autoimmune/Autoinflammatory Skin Diseases—A Focus on Psoriasis and *Lupus erythematosus*

**DOI:** 10.3389/fimmu.2019.01931

**Published:** 2019-08-21

**Authors:** Anna Berekméri, Ana Tiganescu, Adewonuola A. Alase, Edward Vital, Martin Stacey, Miriam Wittmann

**Affiliations:** ^1^Faculty of Medicine and Health, Leeds Institute of Rheumatic and Musculoskeletal Medicine, University of Leeds, Leeds, United Kingdom; ^2^Leeds Biomedical Research Centre, National Institute for Health Research, Leeds Teaching Hospitals, Leeds, United Kingdom; ^3^Faculty of Medicine and Health, Leeds Institute of Cardiovascular and Metabolic Medicine, University of Leeds, Leeds, United Kingdom; ^4^Faculty of Biological Sciences, School of Molecular and Cellular Biology, University of Leeds, Leeds, United Kingdom

**Keywords:** lupus, psoriasis, skin inflammation, tape stripping, hair follicle

## Abstract

The traditional diagnostic gold standard for inflammatory skin lesions of unclear etiology is dermato-histopathology. As this approach requires an invasive skin biopsy, biopsy processing and analysis by specialized histologists, it is a resource intensive approach requiring trained healthcare professionals. In many health care settings access to this diagnostic approach can be difficult and outside emergency cases will usually take several weeks. This scenario leads to delayed or inappropriate treatment given to patients. With dramatically increased sensitivity of a range of analysis systems including mass spectrometry, high sensitivity, multiplex ELISA based systems and PCR approaches we are now able to “measure” samples with unprecedented sensitivity and accuracy. Other important developments include the long-term monitoring of parameters using microneedle approaches and the improvement in imaging systems such as optical coherence tomography. In this review we will focus on recent achievements regarding measurements from non-invasive sampling, in particular from plucked hair and skin tape-strips which seem well suited for the diagnosis of lupus erythematosus and psoriatic inflammation, respectively. While these approaches will not replace clinical observation—they can contribute to improved subgroup diagnosis, stratified therapeutic approaches and have great potential for providing molecular and mechanistic insight in to inflammatory skin diseases.

## Introduction

Traditionally, the diagnosis of autoimmune and inflammatory skin diseases relies on the visual assessment by experienced dermatologists as well as dermato-histopathology. Descriptive diagnosis of skin biopsies is normally based on conventional histological staining but may also include direct immunofluorescence for the detection of antibody and complement deposits. Depending on the underlying disease, blood results in particular regarding circulating auto-antibodies directed against structural epidermal components (pemphigus, pemphigoid) or anti-nuclear antibodies can give certainty regarding the underlying disease. However, as blood focused diagnostics can often be negative for cutaneous disease manifesting, key information must often be derived from analysis of skin tissue itself.

So why are changes or additions to the established diagnostic approach for inflammatory skin disease required? There are a number of reasons. Firstly, due to the non-systemic nature of many skin diseases, reliable serum biomarkers may simply be non-existent or unreliable. Secondly, the use of skin biopsies as a diagnostic tool is an invasive procedure and not always readily available in many health systems or local situations. Skin biopsies require specialized, trained health care professionals, laboratory and histology staff (dermatohistopathologists) and the time to perform biopsies in addition to the wait for result reporting, can in clinical reality often be weeks. Furthermore, as many primary health care settings lack access to specialist dermatology care, approaches suitable for general practitioners (GP) would be very welcome to support diagnostic pathways.

Apart from costs, time and staff availability, an important issue comes from the recognized need and ambition to move forward in the field of stratified medicine (also referred to as personalized medicine, precision medicine). This approach aims to recognize sub-types of diseases outside the traditional morphology based categories, allowing optimized treatment choices and thus preventing the current “trial and error” approach to identify which treatment actually works best for which patient. For this to become a reality for a wide range of patients, researchers, and clinicians need to start thinking of alternatives to conventional approaches and embrace new molecular analysis tools, parameters not traditionally included in dermatology diagnosis, and easy, non-invasive approaches. Ideally, these approaches would not require highly-trained specialists to perform and analyse results, results would be available within the first presentation of the patient (“point-of-care” diagnostic) and would allow repeated diagnostic assessment over time.

While confirming the diagnosis of cutaneous lupus erythematosus (LE) can be challenging due to the wide variety of possible lesion morphologies, the diagnosis of psoriasis is usually directed by pathognomonic clinical presentation. So why is there a need to additional approaches regarding psoriatic inflammation? Diagnostic challenges occur for lesions which do not fall into the typical plaque type psoriasis or display minimal pathology; however, they are important to diagnose due to the existence of co-morbidities such as psoriatic arthritis and cardiovascular disease. Lesions in certain anatomical locations such as palmoplantar, retro-auricular, outer ear canal and scalp can also be challenging for both GP and trained dermatologists, and in particular skin fold lesions can often be misdiagnosed as fungal infection and treated without success with anti-fungals.

In this review we endeavor to give an overview of existing non-invasive techniques and highlight major recent advances demonstrating their potential as next generation diagnostics and as powerful tools to provide cellular and molecular insights in to inflammatory skin diseases.

## Imaging

Recent developments in imaging and imagining analysis techniques have added to our ability to assess real time changes in the skin. The most widely used imaging technique in standard care dermatology for suspicious and/or pigmented lesions is dermatoscopy. Dermatoscopy standards are developed by the international dermatoscopy society (dermoscopy-ids.org) and the approach has also been successfully used to support diagnosis of inflammatory skin conditions [for review: (1–3)]. Dermatoscopy needs training, although machine learning based analysis tools are successfully being developed in particular for cancerous lesions, and is usually performed in dermatology settings.

While this review does not specifically focus on imaging techniques, the following innovative approaches have to be mentioned, although they do require significant financial investments. A recent development, not yet introduced into clinical settings is Raster Scanning Optoacoustic Mesoscopy (RSOM) (4). This technique also termend photoacoustic mesoscopy is based on ultra-broadband (10–180 MHz) detection, achieves tissue resolution of 4 μm axially and 20 μm laterally and importantly can visualize vascular patterns in dermal skin compartments. A number of studies have collected images from human skin including eczema, psoriasis and nail fold changes in scleroderma capillaries (4, 5). This imaging approach can distinguish between intra- and subepidermal morphology features typical for eczema or psoriasis lesions (5).

By contrast, optical coherence tomography (OCT), which can deliver similar morphology information [detailed comparison of OCT vs. RSOM is given in (4)], has been used in a wide range of skin lesions, in particular in neoplastic ones to assess invasiveness and depth of the tumor. OCT uses light to capture sub-micrometer resolution and creates three-dimensional images from upper skin layers. The method is based on low-coherence interferometry employing near-infrared light. The use of relatively long wavelength light allows it to penetrate 1–2 mm into the tissue. This method generates detailed 3D data on skin surface roughness, tissue density, and vascular network in addition to non-invasive measurement of architectural features such as epidermal thickness. This technology is already being utilized as a biomarker for scleroderma and systemic sclerosis (6–8), preclinical diagnosis of palmar hyperkeratosis (9) and to evaluate wound re-epithelialization (10).

In psoriasis, OCT can detect all common psoriasis nail changes including leukonychia/white spots, pitting/localized surface irregularities, diffuse surface waving, onycholysis, and subungual hyperkeratosis (11). Studies evaluating psoriatic plaque vascular morphology are also beginning to surface, although are currently limited by low cohort numbers (12, 13).

In cutaneous LE, OCT has been shown to correlate with histological features of disease including hyperkeratosis, epidermal atrophy, and edema (14). More recently available higher resolution OCT has also been found to be of value for the diagnosis of autoimmune blistering skin diseases. However, to date—this technique is considered auxiliary to skin biopsies in this disease group (15, 16) and larger trials are not available yet.

Unfortunately, at present, costly hardware, expertise and time required to analyzed skin lesions is likely to limit the use of OCT to the research setting and specialist clinics.

Microcirculatory abnormalities are found in many inflammatory skin diseases. High Resolution Laser Doppler imaging (LDI) is a single-probe, non-invasive imaging method that can quantify total local microcirculatory blood perfusion, including capillaries, arterioles, venules, and shunting vessels. Similarly to ultrasound, it is based on the Doppler effect. Briefly, when low-level light is directed onto the skin, a fraction of this light penetrates the skin and interacts with both the static tissue and the moving red blood cells (RBCs). The light that is reflected from this static tissue remains unchanged in wavelength. The light that is scattered from the moving RBCs undergoes a change in wavelength. This backscattered light is then collected by the photodetector of LDI system to provide a signal or reading that is proportional to the speed and density of the moving RBCs (17). Alteration in peripheral blood flow (as measured by LDI) has been shown to correlate with inflammation in skin psoriasis (18). More recently, our group showed that in cutaneous LE, LDI showed better correlation with histology than clinical assessment using the local Cutaneous LE Disease Area and Severity Index (CLASI) or a physician's visual analog scale (19).

## Functional Skin Testing

### Trans-Epidermal Water Loss

Trans-epidermal water loss (TEWL) is a validated measure of skin epidermal permeability barrier function, measured with a non-invasive Tewameter® probe (20, 21). Evaporation of water from the skin occurs as part of normal skin metabolism. As barrier function is disrupted, water loss increases. TEWL measures the density gradient of the water evaporation from the skin, expressed as evaporation rate (g/h/m^2^). In addition to resting TEWL, epidermal function can be assessed by measuring TEWL recovery over time following barrier disruption by repeated tape stripping (20).

Although atopic eczema is best recognized for its barrier defects clinical and molecular evidence suggest that barrier defects play a role in psoriatic disease pathogenesis (22–25). Further, experimental skin barrier disruption leads to several psoriasis-like features including epidermal hyperproliferation, production of pro-inflammatory cytokines, increased inflammatory infiltrate, elevated vascular endothelial growth factor and consequently, increased vascularization (26–29). Conversely, glucocorticoid/corticosteroid therapy reverses many of these features to restore normal barrier function (23, 30). Glucocorticoids also help to maintain the barrier function by directly promoting keratinocyte differentiation pathways (31, 32) which result in a paradoxical improvement in barrier function (33). Interestingly, a recent transcriptomic study found a >90% deficit in expression of the glucocorticoid-activating enzyme 11β-hydroxysteroid dehydrogenase type 1 in psoriatic plaques (34) which has also recently been shown to modulate epidermal barrier integrity (35).

Several small clinical studies have employed TEWL as a measure of treatment efficacy in psoriasis (36–40). However, the use of TEWL as a diagnostic tool has not been studied in detail due to a lack of specificity, as other skin conditions such as eczema also present with impaired epidermal barrier function (41, 42). A recent study by Ye et al. used TEWL to demonstrate differences in barrier recovery between stable and progressive forms of psoriasis in uninvolved skin sites (43). Further studies in larger cohorts are required to fully assess the auxiliary value of TEWL as a non-invasive diagnostic or disease activity measure in psoriasis.

TEWL is mainly examined in diseases with epidermal involvement. Thus, LE which shows inflammatory activity around the basement membrane area may not show significant changes in TEWL but this is currently unexplored.

### Skin Elasticity

Skin elasticity is typically measured by a non-invasive Cutometer® probe (or similar method) which utilizes negative pressure to aspirate a small section of skin. The distance the skin travels is affected by its elastic properties and this is recorded as a series of parameters (44). This has been utilized to measure elasticity in skin disorders characterized by stiffness such as scleroderma and systemic sclerosis (45–47).

A limited number of studies have assessed changes in skin elasticity in psoriasis. Differences in total elastic and plastic deformation were found between psoriasis, dermatitis, and lichen planus patients and within patient groups compared to uninvolved sites, with psoriasis exhibiting the greatest superficial stiffness (48). This could be partly due to psoriasis exhibiting a relatively greater epidermal hyperproliferation and lower hydration than dermatitis and lichen planus (48, 49).

As with TEWL, changes in elasticity were also detected between uninvolved sites in patients with psoriasis and skin from healthy volunteers (50).

Skin elasticity in LE has not been studied in detail but reports indicate differential elastic fiber staining compared to lichen planopilaris which is challenging to distinguish clinically in the later stages (51), suggesting this non-invasive method merits further investigation in LE.

However, the lack of molecular information obtained through this method and variability caused by factors such as age, sun-exposure, anatomical location, and ethnicity are likely to prevent this method being widely used for personalized diagnostics.

## Epidermal Sampling

### Non-invasive Sampling of Skin Epithelium via Tape Stripping

Non-invasive sampling of skin epithelium via tape stripping uses adhesive tapes to “strip” the superficial layers of the skin, peeling of layers tape by tape. This method has been widely used over the last decade in research settings; however with increased sensitivity of detection methods and the given ease of sampling—this approach becomes more interesting for clinical diagnostic pathways.

**Figure 1:**
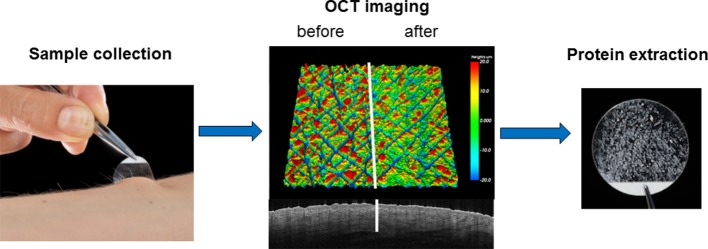
Imaging of a 4 mm volar skin surface area of a healthy forearm with optical coherence tomography (OCT) after application of 50 tape stripes on the right side of the area reveals smoothening of the skin roughness. OCT imaging confirms that sampling of healthy skin remains at the level of the cornified layer and doesn't penetrate deeper than the uppermost layer of the epidermis.

Different types of tapes are available, the most widely used being the acrylic-based (D-Squame discs−1.4 cm diameter, CuDerm, Dallas, TX, USA or 2.2 cm diameter, Monaderm, Monaco), and synthetic rubber-based tapes (Adhesive Research, Glen Rock, PA) but other tapes, such as Barrier (Mölnlycke, Allerod, Denmark) and cellophane tapes (Nichiban Co, Tokyo, Japan) are also utilized. Tapes are placed on the skin with gentle pressure for 2–10 s, and then removed with a quick movement. D-squame pressure applicator (Monaderm) can be used to standardize pressure (225 g/cm^2^). It has been shown that due to the stronger adhesion of corneocytes toward the stratum granulosum, the protein content of tapes decreases in parallel with the increase of tape number/depth of penetration (52, 53). Studies examining the depth of skin removed by tape stripping techniques indicate that in case of healthy skin, depending on anatomical location, at least 30 consecutive tapes are needed to remove the stratum corneum (SC) via D-Squame tapes (54, 55). We have confirmed these results by applying 50 sequential half cut D-Squame discs to a healthy skin area. Simultaneous visualization of the taped and non-taped area was performed with OCT imaging, examining the skin surface roughness and epidermal thickness (Figure 1).

Sample collection can be followed by mRNA or protein isolation from pooled tapes. The composition of buffers for protein extraction differ, however most contain Triton-X100 and protease inhibitor cocktails. Sonication steps can be applied in order to improve the yield of extracted protein.

Skin-taping requires no special preparation of the skin and no anesthetic. In the authors view the largest advantage of tape stripping is that it allows for direct sampling of the pathologically involved skin (lesional skin). In contrast to biopsies, which are not a preferred option in pediatric settings and at certain anatomical sites due to their invasiveness and potential for scar formation, tape stripping can be repeatedly performed at any anatomical site, independent of age and location (56–58).

A potential disadvantage of tape stripping lies in the difficulty of sampling standardization. Quantity and quality of sampled skin may differ not only between individuals, but also intra-individually due to differences in epidermal thickness, skin hydration and intercellular adherence (52, 59–62). A study on RNA yield when sampling healthy skin at different locations demonstrated considerable inter- as well as intra-individual variations (mean RNA mass = 11 ng ± 3.6 from the forearm, and = 91 ng ± 31 from the back, when using 12 consecutive tapes). The authors indicated that rather than corneocytes, other associated cells (specialized keratinocytes, components of adnexal structures) are the main source of RNA recovered by tapes. In addition, a variable distribution in the normal baseline mRNA expression of pro-inflammatory IL-8 and TNFα was demonstrated, which could contribute to differences in disease specific anatomical predilection sites. The study highlights that despite the distinct epidermal characteristics at different sites, mRNA expression can be internally normalized to combined housekeeping gene expression levels, as they are relatively uniformly distributed (63).

Healthy skin sampling is limited to the stratum corneum (SC), however this is often not the case in pathologically involved skin, where inflammation influences not only the morphology [hyperproliferative epidermis with thickened SC in psoriasis vs. reduced SC thickness with increased corneocyte cohesion in acute eczema compared to healthy and non-lesional skin (64)], but also the cellular composition within the epidermis, barrier defects at the level of tight junctions and the basement membrane, thus allowing “leakiness” of serum components into the epidermal compartment. Therefore, the authors believe, that the term “stratum corneum sampling/harvesting” should be avoided when referring to tape stripping in diseased skin, as one cannot be certain which layers of the epidermis are collected. Moreover, additional variations can result from different sampling techniques (e.g., applied pressure, duration, the dynamic of tape removal, precise reapplication at the same site) (61, 65, 66).

Studies focusing on transcriptomic profiling of lesional and non-lesional skin material obtained by non-invasive tape stripping are limited in number. In an early study Morhenn et al. used 20 subsequent acrylic based tapes describing distinct RNA cytokine profiles in allergic and irritant contact dermatitis using ribonuclease protection assay (67). Follow up work by Wong et al. demonstrated that the application of four rubber based tapes is sufficient to obtain adequate amount of RNA, suitable for RT-PCR and amplification for microarray analysis. However, the yield of RNA from healthy skin is low compared to inflamed skin (68). To date, only one study focusing on the “taped” mRNA profile in psoriasis showed an upregulation of TNFα, IFNγ, KRT-16, CD2, IL-23A, IL-12B, and VEGF in psoriatic lesions (69). Interestingly, this study found stronger expression of five of these mRNA levels in tape stripping material compared to punch biopsy material from adjacent sites. This highlights that although tape-striping does not sample the deeper layers of the skin, it may have an advantage in detecting epithelial diagnostic biomarkers in diseases with epidermal involvement.

However, overall there are noteworthy limitations for the use of skin tape-strip sampling for transcriptomic analysis as the quantity and quality of RNA obtained is suboptimal. This is due to the skin surface being particularly rich in RNAases and due to the fact that RNA degradation is a natural process of skin differentiation as cells mature into corneocytes. This makes the method prone to false negative results. A recent study by Dyjack et al. (70) reported an ~40% failure rate in RNA isolation from non-lesional atopic dermatitis (AD) tape samples, compared to the 86% isolation success rate from lesional AD skin. Of interest, transcriptome (RNAseq)—proteome (LC-MS/MS) correlation based on full thickness skin biopsy material from lesional and non-lesional psoriatic skin demonstrated an only modest correlation (71). A clear advantage of focusing on protein detection in diagnostic approaches is their stability and the ease to measure their concentration in a timely manner.

Recent evidence suggests that protein sampling of the epidermis via tape stripping is a surprisingly efficient method to detect and study a range of mediators, from structural proteins to antimicrobial peptides, chemokines, and cytokines contributing to disease pathomechanisms, using traditional ELISA, western blot and Multiplex bead-based technologies where available. Furthermore, studies analyzing protein extracts from tape stripping with highly sensitive mass spectrometry (MS) techniques are emerging as an unbiased approach to examine disease entities not only from the perspective of disease subtype diagnosis, but importantly the detection of differentially expressed proteins can also provide potential mechanistic insight into the initiation and maintenance of these inflammatory diseases.

One of the most comprehensive tape stripping studies focusing on healthy vs. psoriatic skin identified 140 proteins expressed in lesional psoriatic skin by combining tape sampling and MS analysis (72). Compared to healthy, significant upregulation of 20 proteins were found, amongst them proteins playing role in keratinocyte differentiation, antimicrobial activity, cell-cell and cell-matrix interactions, protease inhibition and in signaling pathways (FABP5, fatty acid-binding protein; CALML5, calmodulin-like protein 5; NGAL, neutrophil gelatinase-associated lipocalin, elafin, S100A7 “psoriasin,” α-enolase, galectin-7 (members of the serpin family, 14-3-3-protein sigma). Zn-α-2-glycoprotein was the only protein found to be downregulated in lesional vs. healthy samples. Although promising, many of the mediators found upregulated in psoriatic lesional tape samples compared to healthy have also been described to be also upregulated in tape harvested lesional AD skin, such as galectin-7, Serpin and S100 family members, FABP5 and α-enolase (73–76). Galectin-7 expressed in the SC has been previously studied as an indicator of skin barrier disruption in eczema (77), and serpin B3 has been highlighted as a potential biomarker for atopic inflammation (73, 78), although serpin family members seem to play a pathophysiological role in psoriatic inflammation as well (79, 80).

Additionally, Mehul et al. (72) were able to quantify known pathophysiological chemokines in psoriatic tape samples, including CXCR3 ligands (CXCL10, CXCL11) which are involved in the chemotaxis of IFNγ expressing lymphocytes) and CCL20 which acts on CCR6^+^ IL-17 producing leukocytes (72, 81, 82). Tape sampling of psoriatic skin also detects strong expression of the neutrophil chemoattractants CXCL1 (GROα) and CXCL8 (IL-8) (72, 83). This is in line with the neutrophilic infiltrate known to characterize psoriatic skin lesions (84, 85). Unlike in protein extracts from full thickness biopsies, CXCL1 was also detectable in non-lesional tape samples (72). The same study found low expression but detectable levels of IL-17 pathway cytokines including IL12p40 subunit which is part of both IL-12 and IL-23, IL17A, and IL17F in psoriatic lesional tape samples. According to the authors' experience, in contrast to epidermal chemokines, detection of lymphokines in tape stripped samples from lesional skin is not very reliable probably due to disease activity dependent differences in lymphocyte infiltration into the upper skin layers.

There are a number of studies focusing on atopic inflammation vs. healthy skin which identified elevated CCL17 (TARC) and TSLP in tape harvested material with correlations to disease severity (86, 87). However, none of the mentioned investigations directly compared lesional skin from different diseases which leaves interpretation on whether detected molecules are disease specific or just general inflammation markers open to discussion.

We have recently (88) compared tape harvested proteins expressed in psoriatic and atopic eczema inflammation with comparable lesion scores (e.g., local inflammatory intensity), non-lesional and healthy skin. The study identified increase expression of IL-36γ, a pro-inflammatory IL-1 family member, as a surprisingly robust biomarker for psoriatic inflammation. These results are in line with previous research which showed significantly enhanced IL-36γ mRNA expression in psoriasis biopsy material compared to healthy skin and recognized IL-36γ as an important cytokine in the pathogenesis of psoriatic inflammation (89–91). Other previously described markers including CCL20, CXCL1, and CXCL8, were also strongly increased in lesional psoriatic samples compared to eczema but were not as reliable as IL-36γ in discriminating the two entities.

Lesional psoriatic skin is well described to overexpress antimicrobial peptides (AMPs) in the outermost layers of the skin. Thus, non-invasive surface sampling is ideally suited study their expression in different conditions. AMPs measured via tape stripping (92, 93) but also “washing fluid” (94, 95) obtained from the skin surface confirmed significantly higher expression levels of hBD2 in both lesional psoriatic and eczematous skin compared to healthy controls. To date, there is no published tape stripping data regarding hBD3 expression in psoriasis, although increased secretion of hBD3 and RNase7 have been shown in non-lesional AD compared to healthy skin (96). Markedly elevated S100 family members (S100A7, S100A8, S100A9) expression which contribute to antimicrobial defense but are also an indicator of inflammation at epithelial surfaces, have been reported in psoriatic lesional SC (72). Identification of fecal calprotectin (formed by a heteromeric complex of two subunits, S100A8 and S100A9) is currently used as a routine non-invasive diagnostic tool to distinguish inflammatory bowel disease (IBD) from irritable bowel syndrome, to follow up disease activity and predict disease relapse (97).

All skin diseases showing epidermal inflammation present with some degree of pruritus, which is well recognized to be a complex phenomenon. Neuropeptides which can impact on release of histamine, vasoactive mediators and pro-inflammatory cytokines and play a role in itch. Keratinocytes are the main source of the neurotrophic nerve growth factor (NGF), a regulator of sensory nerves innervating the skin and which can induce lymphocyte activation and keratinocyte proliferation (98, 99). NGF has been recognized to be upregulated in both atopic and psoriatic lesional skin, and correlates positively with the severity of itch (100, 101). A study focusing on AD has shown that NGF is measurable in tape collected samples, and that its levels correlate with disease and itch severity and are found reduced following treatment (102). IL-31 also called the “pruritic cytokine”—which is currently targeted by biologics approach in clinical studies—can be successfully measured in psoriatic lesional SC (72).

Metabolomics is an exciting newly emerging field. Recent data using skin and serum samples suggest that perturbations in the glycolysis and amino acid metabolic pathways may play an important role in the pathogenesis of psoriasis (103, 104). So far there is a lack of studies combining tape stripping with examining metabolomics data, although this could represent a valuable future approach.

### Other Approaches for Epidermal Sampling

Other approaches for epidermal sampling include—as mentioned above—washing buffer approaches which seems to be particularly suitable to monitor the *in vivo* secretion of AMP (94, 95).

Epidermal material can also be obtained by scraping of the skin surface with a surgical blade. For psoriasis lesions, this approach allows detection of chemokines, growth factors including VEGF and IL-1 family members (104). However, this sampling methods seems difficult to standardize.

Suction blister (105) has been successfully used in research settings to allow protein measurement of interstitial fluid from lesional skin—however this approach seems less suitable for clinical practice as it is time intense and requires special setups and training. A promising approach suitable for point of care diagnostic is the analysis of interstitial fluid by means of microneedle patches (106). This approach is being used for continuous glucose monitoring and drug bioavailability. This method has not yet been applied to inflammatory skin conditions.

## Hair Follicle Analysis

Hair follicles are generally referred to as an “appendage” located in the skin (107, 108). Hair biologists however consider the hair follicle an “end organ,” with its own complex microenvironment (109). The hair producing segment of the hair follicle is constantly being renewed from a stem cell pool (110–112). The hair follicle is mainly composed of keratinocytes that make up the hair shaft as well as the inner and outer root sheaths. The hair follicle also has a specialized mesenchymal population referred to as dermal papilla (DP) (112) which play a role in regulating the activities of keratinocytes in forming a follicle and hair shaft. The hair follicle stages include anagen (active growth), catagen (degeneration of the lower follicle), telogen (quiescence), and then regeneration (108, 112).

The bulge region of the hair follicle is located in an area of the outer root sheath just beneath the sebaceous gland and is believed to be the reservoir for epidermal stem cells in the hair follicle (113). This region shows features of an immunologically privileged (IP) site (114). Some of the critical features indicating collapse of immune privilege, which goes along with inflammatory alopecia conditions, is the expression of MCH I and other related molecules, downregulation of CK15, E-cadherin and increased expression of genes associated with epithelial-mesenchymal transition, such as vimentin, fibronectin, and SLUG (115).

### Cutaneous Lupus

Cutaneous lupus can present as an organ specific disease localized only to the skin or can occur as a manifestation of a systemic disease (116). The subtype of CLE that results in permanent scarring is the chronic discoid LE (CDLE) (117). CDLE lesions appear frequently on the scalp, with resultant permanent scar and irreversible hair loss (118, 119). The pathogenesis of CDLE involves accumulation of apoptotic materials, resulting in secondary necrosis and the activation of the interferon (IFN) pathway by nucleic materials, leading to inflammation and the recruitment of cytotoxic, IFNγ-producing CD8^+^ T cells. The recruitment of the cells to the bulge region of the hair follicles coupled with collapse of immune privilege ultimately leads to permanent hair loss and atrophic scarring (120). A key feature in LE is high expression of IFN and IFN stimulated genes (ISGs), such as myxovirus protein A (Mx1), IFN inducible protein 6 (IFI6), guanylate binding protein 1 (GBP-1), CXCL9, and CXCL10 (121–123). In addition to high IFN expression, IFN-induced expression of MHC I and MHC I pathway-related molecule, such as beta 2 microglobulin (β2M) has been reported in *ex vivo* human scalp skin culture (124, 125). IFNs have anti-proliferative properties on skin cells (126) and this may explain the reduced proliferation and neovascularization reported in CDLE cells and tissues, and may be major contributors to the scarring outcome (127).

The current gold standard for diagnosis of connective tissue disease manifesting at the skin organ which includes LE is dermato-immunohistopatholog and key features for LE include interface dermatitis i.e., deposition of inflammatory cells at the dermo-epidermal junction, basal cell vacuolization, keratinocytes apoptosis, lymphohistiocytic infiltration around appendages and vessels, mucin deposition, basement membrane thickening, and follicular plugging (128, 129). An important differential diagnosis for LE of the scalp is Lichen planopilaris (LPP) which also presents as interface dermatitis in dermatohistopathology and can be difficult to distinguish in some cases. As with LE, current knowledge suggests that scarring in LPP is due to CD8^+^ T-cell driven attack on the epidermal stem cells of the hair follicles due to collapse of immune privilege (114).

Scalp dermatoscopy can be a useful addition to the diagnostic portfolio in dermatology settings (130). The hair follicle epithelium has been recognized as a valuable tissue for diagnostic purposes. The group of Blume-Peytavi has demonstrated the diagnostic potential of hair follicles obtained by hair plucking or cyanoacrylate surface stripping in androgenic alopecia and seborrheic dermatitis conditions (131–133).

Since the hair follicle is the main target in CDLE, we used 3–5 plucked anagen hair follicles as a non-invasive diagnostic approach (134). We found increased expression of ISGs that are known to play important role in the pathogenesis of the disease (121). Interestingly, we also found increased expression of β2M. Both of these findings have also been made for LPP by Harries et al. (114) who used laser capture microdissection of scalp biopsy to gain hair follicle specific information (114). Of interest, regarding diagnostic difficulties for CDLE vs. LPP lesion, significant contribution of complement activation identifies LE lesions. In our analysis of plucked hair follicle derived epithelium, we found increased expression of C3 in lesional LE in comparison to non-lesional, psoriatic, and healthy hair follicles.

Of interest, despite the hair follicles not being specifically involved in type 2 diabetes, the use of hair follicles as an alternative means of diagnosing the disease has been suggested (135). This hypothesis is hinged on the prolonged shortage of oxygen supply to the hair follicles in hyperglycaemic patients thus leading to hair follicles damage, sparseness of hair, or decreased hair growth speed (136).

Based on our experience, patients are highly acceptant of hair follicle plucking (in our setup 5–10 hairs) for diagnostic or research purposes and are also happy for repeated sampling e.g., to ascertain therapeutic responses. In addition, hair follicle collection does not require specialist training.

## Conclusion

The need to include analysis of tissue specific responses in precision medicine approaches has been recognized. Table 1 gives an overview including advantages and limitations of the main approaches discussed in this review. Dermatoscopy and functional skin testing are useful to support diagnostic processes but their use seem to be limited to specialized dermatology department and training is needed for correct use and interpretation.

**Table 1:** Characteristics of non-invasive diagnostic approaches.

**Method**	**Current applications**	**Studying**	**Advantages**	**Disadvantages**
**Tape stripping**	Non-invasive sampling of the upper epidermal layers (mainly stratum corneum)	Physiology of the stratum corneum	Non-invasive, no scarring, painless, repeatable, applicable at any anatomical location (e.g., face) and suitable for any age group (including children)	Depth of sampling varies between different skin conditions and different body sites
		Diagnostic markers	Provides valuable molecular information	Need storage access (freezer) and sample transport facility if not processed at site
		Treatment targets	Easy and quick to perform, no special training or professional knowledge is required	
		Disease pathomechanism	Cost effective	
		Disease monitoring	Potential as a non-invasive tool for diagnostic, disease activity and therapeutic response	
		Epidermal wound healing		
		Excretion of endogenous substances		
		Percutaneous absorption of topical treatments—kinetics of drug delivery		
		Treatment efficacy and toxicity (e.g., glucocorticoid therapy)		
	Disruption of the skin barrier	Skin barrier function (TEWL)		
	“Deep” tape stripping = Koebnerisation	Pathophysiology of psoriasis		
**Hair plucking**	Research	Etiology and pathogenesis of diseases involving the hair follicle (LE, LPP, scarring vs. non-scarring alopecias)	Relatively non-invasive, repeatable, and suitable for any age group (including children)	Variability in the quality of hair follicle obtained
		Wound healing	Provides valuable molecular information	Restricted to patients with presence of “pluckable” hairs; so far only investigated for the scalp
		Therapeutic response	Easy and quick to perform, however, the examiner needs to be able to differentiate anagen from telogen hair	Need for further scientific data
			Potential as a non-invasive tool for diagnostic, disease activity and therapeutic response	Results may depend on hair cycle stage. The same hair cycle stage should be analyzed (e.g., anagen) depending on research question
				Need storage access (freezer) and sample transport facility if not processed at site
**Trans-Epidermal Water Loss (TEWL)**	Skin epidermal permeability/barrier function	Barrier defects in various pathologies (eczema, psoriasis)	Non-invasive, painless, repeatable, applicable at any anatomical location (e.g., face) and suitable for any age group (including children)	Lack of molecular information—lack of specificity as a diagnostic tool
		Treatment efficacy and toxicity (e.g., glucocorticoid therapy)	Validated measure, quick, and accurate	Need access to the instrument (Tewameter®) and specific software
				Application limited to epidermal pathologies
**Skin elasticity**	Skin elasticity measurements	Skin disorders characterized by stiffness (scleroderma, systemic sclerosis) and, other pathologies	Non-invasive, painless, repeatable, applicable at any anatomical location (e.g., face) and suitable for any age group (including children)	Lack of molecular information—lack of specificity as a diagnostic tool
		Skin aging		Variability (age, sun-exposure, anatomical location, ethnicity)
		Treatment efficacy and toxicity (e.g., glucocorticoid therapy)		Need access to the instrument (Cutometer®) and specific software

As highlighted in this review, novel imaging techniques have great potential to allow non-invasive diagnostic by visualizing changes in the epidermal down to the vessel compartment. Access to imaging devices such as OCT and RSOM are however limited to specialized centers due to high costs.

By contrast, epidermal sampling by tape stripping and for the scalp by hair follicle plucking do not need specific training, are easy and fast to perform, suitable for point of care approaches and can give molecular information beyond morphology changes. This could significantly help with disease subgroup identification and importantly provide a molecular understanding of the inflammatory processes behind these diseases and highlight novel targets for future therapeutics. Thus, protein biomarkers detectable with non-invasive epidermal/hair follicle sampling has the potential to direct treatment pathways, follow up disease progression and therapeutic response.

It remains to be demonstrated in well planned prospective studies which compare standard of care diagnostic with the here discussed non-invasive diagnostic approaches if suitable protein biomarker algorithm can be developed with high enough sensitivity and specificity for a range of inflammatory skin conditions to replace current biopsy approaches. Another previously discussed (137) outcome of such studies could be that approaches such as tape stripping cannot fully replace dermatohistopathology but could prove to be of significant support for treatment decision pathways either in primary care setting (e.g., inverse psoriasis vs. fungal infection; palmoplantar eczema vs. psoriasis), in secondary care dermatology (e.g., predicting response to therapy) or in other disciplines such as rheumatology (e.g., supporting the diagnosis of psoriatic arthritis). The potential of epidermal sampling to be developed into a point-of-care test for specific diagnostic problems is given by the ease of sampling. Larger trials and development of robust biomarker algorithms are needed to fully appreciate the value epidermal sampling could have in routine care settings.

## Author Contributions

All authors contributed to writing the manuscript. AB performed the optical coherence tomography imaging shown in Figure 1.

### Conflict of Interest Statement

The authors declare that the research was conducted in the absence of any commercial or financial relationships that could be construed as a potential conflict of interest.
